# A challenging diagnosis of dermatofibrosarcoma protuberans of the middle finger in an infant: A case report

**DOI:** 10.1016/j.ijscr.2024.109890

**Published:** 2024-06-08

**Authors:** Majd Hanna, Abdulrahman Saad Alden Alkhatib, Riffa Alassri, Rim Awada, Dalaa Daboura, Nafiza Martini

**Affiliations:** a Damascus university, Faculty of Medicine, Damascus, Syrian Arab Republic; b Stemosis for Scientific Research, Damascus, Syrian Arab Republic; c Aleppo University, Faculty of Medicine, Aleppo, Syrian Arab Republic; d Hama University, Faculty of Medicine, Hama, Syrian Arab Republic; e Faculty of Medical Sciences, Lebanese University, Rafic Hariri University Campus, Hadath, Lebanon

**Keywords:** Dermatofibrosarcoma protuberans, Middle finger, DFSP, Challenging diagnosis, Case report

## Abstract

**Introduction:**

Dermatofibrosarcoma protuberans (DFSP) is a rare sarcoma, accounting for less than 0.1 % of tumors. While it predominantly occurs in adults, pediatric cases are unusual. This case report aims to highlight the diagnostic and therapeutic challenges posed by DFSP in infants due to its rarity and slow-growing nature, emphasizing the importance of early diagnosis and prompt intervention.

**Case presentation:**

We report the case of an 8-month-old infant presenting with a progressive finger mass, initially mistakenly diagnosed as a dermatofibroma. Local excision was done, but the tumor recurred after one year. Subsequent re-excision and skin grafting were performed, and histopathology confirmed DFSP. Despite middle finger amputation three weeks later, a new mass emerged on the adjacent ring finger after one year. This tested negative for DFSP. The fibrous mass has persisted for five years without significant changes.

**Clinical discussion:**

DFSP is a rare sarcoma with a higher prevalence in adults. It typically presents as a painless, slow-growing mass and is usually diagnosed by biopsy and immunohistochemistry. Surgical excision with negative margins is the preferred treatment. The rarity and slow-growing nature of DFSP pose challenges in diagnosis and treatment.

**Conclusion:**

Early diagnosis and prompt surgical intervention are crucial in managing DFSP, especially given its high recurrence potential. Maintaining a high index of suspicion is essential even in very young children. Aggressive resection with negative margins and diligent post-operative surveillance are key strategies to mitigate metastasis risk and improve prognosis in such challenging cases.

## Highlights

• DFSP is a rare type of skin sarcoma that typically affects people aged 20-40 years old and rarely pediatric patients.
• DFSP often starts as a painless, slow-growing skin mass, but can develop into a large protuberant mass.
• Diagnosis is through biopsy and immunohistochemistry. Treatment is surgical excision with clear margins.
• Recurrence rates are high with simple excision so more aggressive removal is preferred.

## Abbreviations

DFSP: Dermatofibrosarcoma Protuberans
MMS: Mohs micrographic surgery
PDGF: Platelet-derived Growth Factor
WHO: World Health Organization

## 1 Introduction

Dermatofibrosarcoma Protuberans (DFSP), is a rare sarcoma that typically affects individuals between the 2nd and 4th decades of life, with a slightly higher prevalence among females [1,2]. DFSP can present as a painless, slow-growing mass that gradually develops into a hard, large, protuberant lesion, rarely spreading to regional lymph nodes or metastasizing. It accounts for approximately 1 % of all soft tissue sarcomas [3].

Diagnosis is often made through biopsy and the application of immunohistochemistry. DFSP has two main treatment approaches: resectable and unresectable. Resectable DFSPs are treated by complete surgical excision, either through wide local excision with tumor-free margins or Mohs micrographic surgery (MMS). In rare cases, amputation may be necessary [4]. However, recurrence rates can reach up to 50 % after basic excision procedures. To reduce recurrence rates to 5 % or lower, more extensive resection and thorough consideration of radial margins are essential [5]. Unresectable DFSPs are typically treated with radiation therapy and/or targeted therapy [4].

The rarity of this sarcoma, along with its clinical characteristics and slow-growing nature, can pose challenges for clinicians in terms of diagnosis and treatment. In this particular case, we report a patient who was initially misdiagnosed with dermatofibroma, which later turned out to be Dermatofibrosarcoma Protuberans (DFSP). This initial misdiagnosis likely contributed to the recurrence of the tumor, requiring a more aggressive treatment approach. This work has been reported in line with the SCARE criteria [6].

## 2 Case presentation

An 8-month-old girl was brought to the clinic by her parents, concerned about a growing lump on her middle finger that had initially appeared when she was 3 months old. The lump started as a small, firm, and painless mass, but no medical attention was sought at that time. Upon the lump's growth in size, the child was evaluated, revealing a 2-cm non-painful lesion on the distal phalanges of the middle finger. The lesion had a glossy appearance, firm consistency, well-defined margins, and no signs of inflammation or nodular enlargements elsewhere on the body. A biopsy initially diagnosed the mass as a Dermatofibroma, which was subsequently excised with local excision and regular monitoring.

**Fig. 1:**
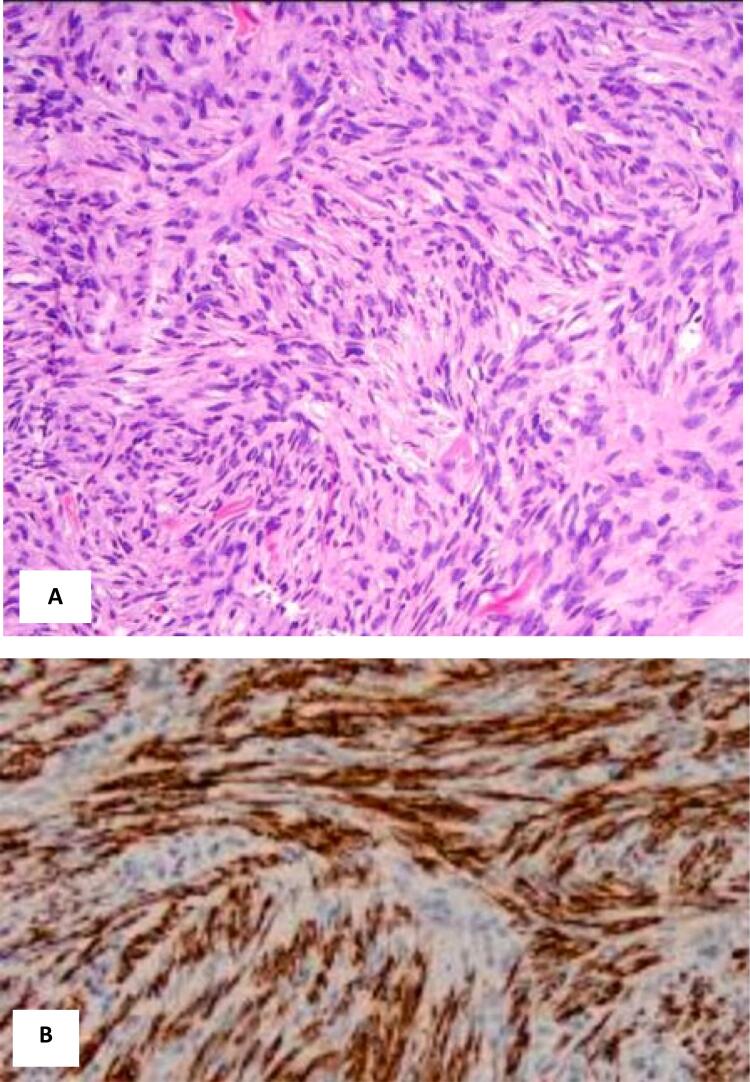
Histopathologic and immunohistochemical findings. (A) Hematoxylin and eosin stain showing proliferation of spindle cells with oval, vesicular nuclei, eosinophilic cytoplasm and low mitotic activity. (B) Immunohistochemical analysis revealing diffuse strong immunoreactivity for CD34 in the spindle cell population.

However, one year later, the patient returned, reporting the reappearance of the tumor, which had been gradually growing in size. The tumor was entirely excised, with careful clearing of the surrounding margins, and a skin graft was taken from the neighboring ring finger, which was fused with the affected finger. The excised tissue was examined pathologically, revealing a tumoral proliferation invading the dermis and subcutaneous tissue, and strongly positive CD34 immunohistochemical staining. This second biopsy diagnosis indicated the presence of Dermatofibrosarcoma protuberance (Fig. 1).

**Fig. 2:**
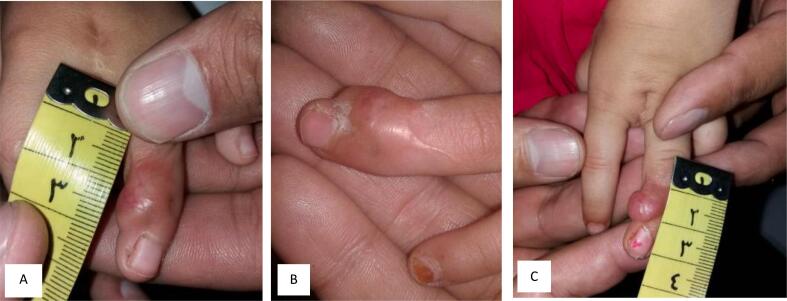
Images (A, B, C) showing a lump gradually increasing in size on the fourth finger.

**Fig. 3:**
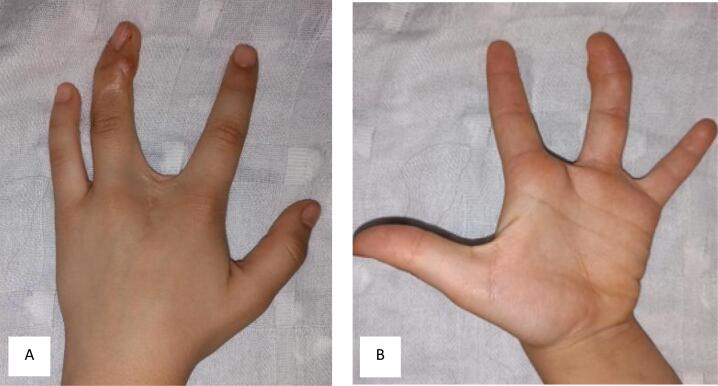
Images (A, B) showing the child's hand following a five-year period of observation.

The initial misdiagnosis of Dermatofibroma may have contributed to the recurrence of the tumor, as Dermatofibrosarcoma protuberance is more aggressive and known to have a high local recurrence rate. Accordingly, three weeks later, the fingers were separated, and the middle finger was surgically removed due to concerns about possible unresected metastases. Ongoing monitoring was conducted for the fourth finger. Later, during the year, a fresh lump was detected on the fourth finger, displaying a gradual increase in size (Fig. 2). A biopsy of this emerging tumor revealed the presence of granulation tissue with reactive fibrous changes, and the tumor tested negative for CD34, indicating the absence of tumor metastasis. The fibrous mass in the ring finger persists, measuring 1 cm in size, and has remained unchanged for the past five years and up to the present time (Fig. 3).

## 3 Discussion

Dermatofibrosarcoma protuberance (DFSP) is an uncommon medical condition, accounting for less than 0.1 % of all tumors and 1.8 % of all soft tissue sarcomas. It is even rarer in the pediatric population, with an estimated incidence of one per million in individuals under 20 years old [1,7]. DFSP is classified by the World Health Organization (WHO) as a low-grade sarcoma due to its localized aggressiveness and low tendency to metastasize [2]. The transformation of DFSP into a high-grade sarcoma is extremely infrequent. Although certain studies suggest a slightly higher occurrence among females, the research findings remain inconclusive [3,8]. Although DFSP typically affects patients between 20 and 50 years of age, it has been documented in individuals of all age groups, including congenital cases [4]. In our case, the patient was a female in her first year of life, representing the most unusual age group affected by this condition.

The etiology of DFSP remains unclear, although certain risk factors have been proposed, such as local skin injury in the affected area. DFSP has been observed to develop in areas with previous burn wounds, surgical scars, and sites of multiple immunizations [9]. However, no specific distinctions were identified in the medical history of our patient. In terms of genetic factors, studies have indicated a chromosomal translocation t(17;22)(q22;q13) between chromosome 17 and chromosome 22. This translocation leads to the formation of a fusion protein known as COL1A1-PDGFB, which stimulates tumor growth by promoting the excessive production of platelet-derived growth factor (PDGF) [5,10]. However, we cannot afford this genetic testing due to bad economic situation in Syria.

DFSP commonly occurs in specific anatomical locations, with the trunk being the most frequent site (50 %), followed by the proximal extremities (35 %), and the head/neck region (15 %). However, it rarely affects the distal extremities such as the hands, fingers, or feet below the knees [4,11]. In our case, DFSP was located on the middle finger. Typically, DFSP manifests as an asymptomatic, skin-colored to red-brown hardened plaque, which eventually develops multiple raised nodules ranging in color from violaceous to red-brown. The growth of these nodules is slow, and they can attain several centimeters in diameter [12]. Initially, patients may mistake the tumor, especially in its early stages, for a benign lesion such as a keloid or scar. The progression of DFSP is gradual and extensive, and it does not respond to typical local symptomatic treatments. Changes in the tumor's appearance or the occurrence of symptoms like ulceration, infection, or bleeding prompt patients to seek medical attention [13]. Consequently, diagnostic delays are common in such cases. In the present report, the parents noticed the lesion when the baby was three months old. However, the asymptomatic nature of the lump and its indolent behavior deterred them from seeking medical help. This delay may be attributable to financial constraints, particularly among residents of low- and middle-income countries such as Syria, a developing country. Although there may be a delay in diagnosing dermatofibrosarcoma protuberans (DFSP), the occurrence of distant metastasis is uncommon, accounting for less than 2–4 % of cases [14].

The recommended treatment for DFSP is complete resection with a wide margin of surrounding tissue, including the underlying fascia. It is crucial to achieve a safety margin that extends 3 to 4 cm beyond the visible tumor, and the resection margins must be histologically negative, Mohs micrographic surgery (MMS) is considered the gold standard for achieving a cure in cases of skin cancer, and it is among the surgical procedures employed for this purpose [15]. If the margins are positive, the likelihood of local recurrence is approximately 70 % of patients [16]. This highlights the importance of performing a more aggressive resection and carefully assessing the radial margins during the initial intervention. This approach leads to a reduction in recurrence rates to as low as 5 % or even lower, as recurrent cases often require additional surgeries that can hinder the likelihood of achieving primary wound closure [17]. In our case, recurrence occurred one year after the initial excision, likely because of the margin-positive resection. Due to the unpredictable subclinical extension of DFSP, there was concern about tumor spread to adjacent fingers. Consequently, in our case, radical removal of the middle finger was performed to mitigate the risk.

Due to the possibility of recurrence as well as metastasis, close surveillance is imperative, with evaluation every 3 to 6 months for the first three post-treatment years and at minimum on an annual basis thereafter [18]. Our patient has been under follow-up for five years since the recurrence, and at the time of writing, there have been no signs of tumor metastasis.

## 4 Conclusion

Dermatofibrosarcoma protuberans (DFSP) presents unique challenges due to its rarity and diverse clinical presentations across all age groups, including pediatric cases. Early diagnosis is critical for initiating timely treatment and improving outcomes. Treatment complexities stem from the necessity of wide-margin complete resection, emphasizing meticulous surgical planning and margin evaluation. Genetic testing guides personalized treatment strategies, while multidisciplinary care involving dermatologists, oncologists, surgeons, and pathologists optimizes patient management. Ongoing research and surveillance are essential for refining treatment approaches and ensuring long-term outcomes. A comprehensive approach is vital for addressing DFSP's complexities and achieving optimal patient care, driving advancements in its management.

## Consent

Written informed consent was obtained from the patient's parents for publication of this case report and any accompanying images. A copy of the written consent is available for review by the editor of this journal on request.

## Authorship

All authors attest that they meet the current ICMJE criteria for authorship.

## Ethical approval

Our institution does not require ethical approval for reporting individual cases or case series.

## Funding

The authors received no financial support for the research, authorship, and/or publication of this article.

## Author contribution

**Majd Hanna is the first author,** contributed to drafting, editing & reviewing. The author reviewed and accepted the paper.

**Abdulrahman saad alden alkhatib** contributed to drafting, reviewing, & editing. The author reviewed and accepted the paper.

**Riffa Alassri** contributed to drafting, editing, & reviewing. The author reviewed and accepted the paper.

**Rim Awada** contributed to drafting, editing, & reviewing. The author reviewed and accepted the paper.

**Dalaa Daboura** contributed to drafting, editing, reviewing, & bibliography. The author reviewed and accepted the paper.

**Nafiza Martini is the supervisor,** contributed to editing, reviewing & mentorship. The author reviewed and accepted the paper.

## Guarantor

Nafiza Martini accepts full responsibility for the work, had access to the data, and controlled the decision to publish.

## Research registration number

Not Applicable since it is a case report.

## Conflict of interest statement

The authors declare that they have no known competing financial interest or personal relationship that could have appeared to influence the work reported in this paper.
